# Author Correction: Ultra-thin lithium aluminate spinel ferrite films with perpendicular magnetic anisotropy and low damping

**DOI:** 10.1038/s41467-023-43051-2

**Published:** 2024-01-15

**Authors:** Xin Yu Zheng, Sanyum Channa, Lauren J. Riddiford, Jacob J. Wisser, Krishnamurthy Mahalingam, Cynthia T. Bowers, Michael E. McConney, Alpha T. N’Diaye, Arturas Vailionis, Egecan Cogulu, Haowen Ren, Zbigniew Galazka, Andrew D. Kent, Yuri Suzuki

**Affiliations:** 1 Department of Applied Physics, Stanford University, Stanford, CA 94305 USA; 2 Geballe Laboratory for Advanced Materials, Stanford University, Stanford, CA 94305 USA; 3 Department of Physics, Stanford University, Stanford, CA USA; 4 National Institute of Standards and Technology, Gaithersburg, MD 20899 USA; 5 Air Force Research Laboratory, Wright Patterson Air Force Base, Dayton, OH 05433 USA; 6 Advanced Light Source, Lawrence Berkeley National Laboratory, Berkeley, CA 94720 USA; 7 Stanford Nano Shared Facilities, Stanford University, Stanford, CA 94305 USA; 8 Department of Physics, Kaunas University of Technology, Studentu Street 50, LT-51368 Kaunas, Lithuania; 9 Center for Quantum Phenomena, Department of Physics, New York University, New York, NY 10003 USA; 10 Leibniz-Institut für Kristallzüchtung, Max-Born-Str. 2, 12489 Berlin, Germany

**Keywords:** Materials for devices, Magnetic properties and materials, Surfaces, interfaces and thin films, Spintronics, Spintronics

Correction to: *Nature Communications* 10.1038/s41467-023-40733-9, published online 15 August 2023

The original version of this Article contained an error in the author affiliations. Lauren J. Riddiford was incorrectly associated with ‘Paul Scherrer Institut, Forschungsstrasse 111, 5232 Villigen, Switzerland’. This has now been corrected in both the PDF and HTML versions of the Article.

The original version of this Article contained an error in the author affiliations. Egecan Cogulu and Haowen Ren were incorrectly associated with ‘Department of Physics, New York University, New York, NY 10003, USA’. This has now been corrected in both the PDF and HTML versions of the Article.

The original version of this Article omitted the following from the Acknowledgements: H.R. was supported by Quantum Materials for Energy Efficient Neuromorphic Computing (Q-MEEN-C), an Energy Frontier Research Center funded by the US Department of Energy (DOE), Office of Science, Basic Energy Sciences (BES), under Award DE-SC0019273. E.C. and A.D.K. were supported by the National Science Foundation under Award DMR-2105114. This has now been corrected in both the PDF and HTML versions of the Article.

