# Supplementary material for: Ultra-thin lithium aluminate spinel ferrite films with perpendicular magnetic anisotropy and low damping
Source: Nat Commun. 2023 Aug 15;14:4918. doi: 10.1038/s41467-023-40733-9 (PMC10427713; doi:10.1038/s41467-023-40733-9)
Supplement: Supplementary file 1 — Supplementary Information [file 41467_2023_40733_MOESM1_ESM.pdf]

SUPPLEMENTARY MATERIALS
Ultra-Thin Lithium Aluminate Spinel Ferrite Films with Perpendicular Magnetic Anisotropy and Low Damping

Xin Yu Zheng*[1,2], Sanyum Channa*[1,3], Lauren J. Riddiford[4], Jacob J. Wisser[5], Krishnamurthy Mahalingam[6], Cynthia T. Bowers[6], Michael E. McConney[6], Alpha T. N'Diaye[7], Arturas Vailionis[8,9], Egecan Cogulu[10], Haowen Ren[10], Zbigniew Galazka[11], Andrew D. Kent[12], and Yuri Suzuki[1,2]

[1]*Geballe Laboratory for Advanced Materials, Stanford University, Stanford, California 94305, United States*
[2]*Department of Applied Physics, Stanford University, Stanford, California 94305, United States*
[3]*Department of Physics, Stanford University, Stanford, California 94305, United States*
[4]*Paul Scherrer Institut, Forschungsstrasse 111, 5232 Villigen, Switzerland*
[5]*National Institute of Standards and Technology, Gaithersburg, Maryland 20899, USA*
[6]*Air Force Research Laboratory, Material and Manufacturing Directorate, Wright-Patterson AFB, OH 45433, USA*
[7]*Advanced Light Source, Lawrence Berkeley National Laboratory, Berkeley, California 94720, USA*
[8]*Stanford Nano Shared Facilities, Stanford University, Stanford, California 94305, USA*
[9]*Department of Physics, Kaunas University of Technology, Studentu Street 50, LT-51368 Kaunas, Lithuania*
[10]*Department of Physics, New York University, New York, New York 10003, USA*
[11]*Leibniz-Institut für Kristallzüchtung, Max-Born-Str. 2, 12489 Berlin, Germany*
[12]*Center for Quantum Phenomena, Department of Physics, New York University, New York, New York 10003, USA*

*These authors contributed equally to this work.

1 X-ray diffraction

In Fig. S1 (a)-(d) we show X-ray diffraction symmetric scans around the LAFO (004) Bragg peaks for four different LAFO film thickness. We fit each spectra using a dynamical XRD model with the in-plane lattice constant of LAFO clamped to that of the MGO substrate. This coherent growth is evident from the lack of film relaxation observed from reciprocal space maps. The fits yield the out-of-plane lattice constant c as well as the thickness of the strained LAFO films. The thicknesses were further verified using X-ray reflectivity. In Fig. S1 (e) we plot the extracted c-axis values for LAFO across different thicknesses. There is no appreciable trend with thickness indicating uniform tetragonal distortion across thicknesses. The c-axis lattice constant for the films of $\sim$8.1 Å is lower than the bulk LAFO value (determined from XRD on the ablation target) of 8.18 Å, which is consistent with tetragonal distortion resulting from a 1.2% in-plane tensile strain. This tensile strain is also the origin of the PMA due to magnetoelastic coupling (see Supplemental Material Section 3), and a similar effect has been observed in other ferrimagnetic insulator thin films [1, 2, 3]. Rocking curves on the film and substrate (004) peaks shown in the insets of Fig. S1 (a)-(d) exhibit identically low full-width-half-maximum of $\Delta\omega_{004,\mathrm{FWHM}} \approx 0.003°$, indicating low mosaicity and excellent film quality.

Fig. S2 shows a X-ray reflectivity scan on a Pt(15 nm)/LAFO(15 nm)/MGO sample, showing prounced Kiessig fringes indicating a smooth interface. AFM performed on the MGO and LAFO surfaces have typical RMS roughness less than 0.1 nm.

2 Static magnetic characterization

Fig. S3 (a)-(d) shows field dependent magnetization loops for four LAFO film thicknesses. All thicknesses exhibit PMA with [001] (and related in-plane directions) as the easy axis and coercivities as low as 0.6 mT for the 4.1 nm film. The origin of the PMA can be understood in terms of a magnetoelastic contribution to the magnetic anisotropy. Let us assume bulk values of the magnetocrystalline anisotropy and magnetostriction constants, $K_1 = -9.0 \times 10^3$ J/m^3, $K_2 = 2.1 \times 10^3$ J/m^3, $\lambda_{100} = -2.6 \times 10^{-5}$, $\lambda_{111} = 2.3 \times 10^{-6}$ [4]. In the absence of strain effects, the shape anisotropy dominates over the magnetocrystalline anisotropy and favors easy-plane magnetism. However, the lattice mismatch between LAFO and MGO results in an in-plane bi-axial tensile strain ϵ of about 1.2% on the LAFO. Assuming a typical value of $Y \approx 1.25 \times 10^{11}$ J/m^3 for the Young's modulus for bulk LFO [5], the elastic energy density is on the order of $\epsilon Y \lambda \approx 4 \times 10^4$ J/m^3, which is several times larger than the magnetocrystalline anisotropy and an order of magnitude larger than the shape anisotropy $U_{\text{shape}} = \mu_0 M_s^2/2 \approx 3.5 \times 10^3$ J/m^3. A standard energy calculation shows that this large energy scale favors the moments to lie out-of-plane [6], which is consistent with our observation of PMA in tensilely strained LAFO films. This will be further discussed in section 3. In Fig. S3 (e), we plot the net saturation moment per unit area (equivalently $M_s t$) against thickness. The relationship is linear with a negligible y intercept, indicating that LAFO films do not exhibit magnetic dead layers generally found in other FMI thin film systems [7, 8], corroborating the TEM data in the main text. The error bars on M_s were estimated from the linear fits of the diamagnetic background from the substrate that were subtracted from the signal in the SQUID measurements (the intercept term). This error bar is typically on the order of 1%. The film area was determined by overlaying a transparent grid with 1 mm spacings on top of the sample and calculating the area of a (possibly irregular)

polygon constructed from the sample borders. We estimate the errors of this method to be about 10%. Finally, the thickness was determined by fitting the XRD and XRR spectra of the films that has an uncertainty of less than 0.1 nm. The total uncertainty of the net magnetization is calculated by multiplying these quantities and propagating the uncertainties for each. The absence of a dead layer minimizes the thickness of the necessary FMI layer in heterostuctures, making LAFO ideal for a variety of applications.

3 Broadband ferromagnetic resonance

For pure Gilbert-like damping, the ferromagnetic resonance (FMR) linewidth ΔH_{hwhm} scales linearly with frequency f according to [9, 10]:

$$\Delta H_{\text{hwhm}} = \Delta H_0 + \alpha \frac{h}{g\mu_0\mu_B} f, \tag{E1}$$

where α is the Gilbert damping paramenter, g is the Landé g-factor ($g \approx 2.00$, see Fig. S4), h is the Planck's constant, μ_0 is the permeability of free space, μ_B is the Bohr magneton, and ΔH_0 is the inhomogeneous linewidth. Performing a linear fit to ΔH_{hwhm} as a fuction of f thus allows us to extract α and ΔH_0

Fig. S4 (a) shows out-of-plane H_{fmr} as a function frequency f for the four LAFO film thicknesses. For PMA systems the dependence of the FMR resonance field H_r and frequency f follows the out-of-plane Kittel equation [11]

$$f = \frac{g\mu_B\mu_0}{h}(H_{fmr} - M_{eff}), \tag{E2}$$

where $M_{eff} = H_\perp + M_s$ is the effective magnetization, containing the out-of-plane uniaxial anisotropy field $H_\perp$ and the saturation magnetization M_s (here we define

negative M_{eff} to favor PMA). By fitting the data to equation (E2), we can extract the g-factor and M_{eff}. The shift of the FMR frequency with sample thickness as seen in Fig. S4 (a) is consistent with the change of PMA strength in Fig. S3. Fig. S4 (b) shows the effective out-of-plane uniaxial anisotropy energy density $K_{\text{eff},\perp}$ and g-factor from fitting the data in (a). Evidently the PMA strength decreases for lower thicknesses. Given that the films exhibit similar c-axis values across thicknesses (Fig. S1 (e)) and no magnetic dead layer (Fig. S3 (e)), it is unlikely that the change in anisotropy is due to differences in strain state or stoichiometry. Similar behavior has been observed in other PMA thin film systems, and are attributed to interface effects [12, 13, 14, 15]. We also observe a g value close to the free electron value of 2.0 across all thicknesses, consistent with the $3d^5$ electronic configuration of Fe^{3+} with $L = 0$.

Fig. S5 (a)-(d) shows out-of-plane FMR linewidth as a function of frequency for the four film thicknesses. Note that due to increasing anisotropy with thickness, thicker films have an increasing range of low frequencies that are inaccessible for FMR. Fig. S5 (e) shows the extracted Gilbert damping parameter α across thicknesses. The lowest damping achieved is $\alpha = (6.4 \pm 0.6) \times 10^{-4}$ for the 15.1 nm film. In Fig. S5 (f), ΔH_0 (the y intercept of the linear fits in (a) - (d)) is plotted as a function of film thickness. This intrinsic linewidth contribution is attributed to magnetic inhomogeneity and mosaicity in the film, and takes the form [11, 16, 17]

$$\Delta H_0 = \left| \frac{\partial H_{fmr}}{\partial \theta_H} \right| \Delta\theta_H + \left| \frac{\partial H_{fmr}}{\partial M_{\text{eff}}} \right| \Delta M_{\text{eff}}, \tag{E3}$$

where H_{fmr} is the FMR resonance field, θ_H is the angle H makes with the c axis (film normal), $\Delta\theta_H$ is the mosaic spread in c, and ΔM_{eff} is the variation in the effective magnetization $M_{\text{eff}} = M_s + H_\perp$ in the film, where M_s is the saturation magnetization and $H_\perp$ is the uniaxial anisotropy. The two derivatives are to be calculated from the

Kittel equation [11]

$$\left(\frac{fh}{g\mu_B\mu_0}\right)^2 = \left[H_{fmr} - M_{\text{eff}}\cos(2\theta_H) + H_{\text{cub}}(3\sin^2\theta_H\cos^2\theta_H - \cos^4\theta_H)\right] \times$$
$$\left[H_{fmr} - M_{\text{eff}}\cos^2\theta_H - H_{\text{cub}}\cos^4\theta_H\right], \tag{E4}$$

where h is the Planck's constant, f is the resonance frequency, μ_B is the Bohr magneton, and H_{cub} is the magnetocrystalline cubic anisotropy. Computing the derivatives for the out-of-plane geometry ($\theta_H = 0$) yields $\partial H_{fmr}/\partial\theta_H|_{\theta_{\text{H}}=0} = 0$ and $\partial H_{fmr}/\partial M_{\text{eff}}|_{\theta_{\text{H}}=0} = 1$, so that we have $\Delta H_0 = \Delta M_{\text{eff}}$. The results of Fig. S5 (f) thus suggest a typical $\mu_0\Delta M_{\text{eff}}$ of 1-2 mT, which is small compared to $|\mu_0 M_{\text{eff}}| \geq 100$ mT, indicating that the films have excellent magnetic uniformity.

4 Angular dependent magnetoresistance

In Fig. S6 (a), we show angular dependent magnetoresistance (ADMR) measurements on patterned Pt(2 nm)/LAFO(4.1 nm) film at $\mu_0 H = 1$ T. Contributions to the overall magnetoresistance include the proximity induced anisotropic magnetoresistance (AMR) and the spin Hall magnetoresistance (SHMR) due to the transfer of spin angular momentum across the Pt/LAFO interface. These two sources of magnetoresistance can be disentangled by rotating H in planes perpendicular to the sample as shown in the blue and red data in Fig. S6 (a) [18,19,20]. When H is large enough to fully align the moments along H, the AMR and SHMR each follow a cosine squared dependence [21]. From fitting, we extract the magnitudes $|\Delta\rho_{\text{SHMR}}| = 682 \pm 2\ m\Omega$ and $|\Delta\rho_{\text{AMR}}| = 14 \pm 1\ m\Omega$, indicating that the primary source of magnetoresistance in our system is due to the transfer of spin angular momentum across the interface. From the increase in damping with the Pt layer, we estimate that the spin mixing conductance at the interface is approximately $3 \times 10^{14}\ \Omega^{-1}m^{-2}$. We do note that our

AMR signal shows the opposite sign as that expected by MPE. For MPE induced AMR, the resistance is low (high) when the field is perpendicular (parallel) to the current. Our AMR has the opposite behavior, with resistance high (low) when the field is perpendicular (parallel) to the current. In the literature this opposite behavior is attributed to ordinary magnetoresistance (OMR) [22]. While the behavior does not signify the complete absence of MPE induced AMR, it does suggest that the MPE contribution is sufficiently small to be dominated by OMR.

For SHMR, the longitudinal magnetoresistance is high (low) when the moments in the magnet are perpendicular (parallel) to the spins accumulated at the interface from the spin Hall effect. In Fig. S6 (b) we show magnetoresistance as a function of in-plane fields parallel and perpendicular to the current. For a current along x (geometry shown in insets of Fig. S6), the accumulated spins at the interface are parallel to y. When a field is swept along x, the PMA causes the moments to rotate in the xz plane. Since these moments are always perpendicular to y, we do not see an appreciable change in the resistance. In contrast, when the field is swept along y, the moments rotate in the yz plane. At large $|H_y|$, the moments point along $\pm y$ and are parallel to the accumulated spins, resulting in a lower resistance. When $|H_y|$ is small the PMA pulls the moments to point along z, being perpendicular to y which results in a higher resistance. The peak half-width of the H_y data therefore provides an estimate of the effective uniaxial anisotropy field $|\mu_0 M_{\text{eff}}|$. In this case it is about 30 mT, much lower than the value of $|\mu_0 M_{\text{eff}}| \approx 110$ mT obtained from FMR (Fig. S4). This discrepancy is unlikely due to spatial non-uniformity of the film as the anisotropy measured on multiple Hall bars on the same sample are very similar. On thicker samples (which have more negative M_{eff}), we also observe a similar discrepancy of $50 - 70$ mT in the anisotropy after patterning. This suggests that the

change in anisotropy is due to the Pt overlayer, or the patterning procedure itself caused by additional geometrical factors introduced by the Hall bar shape. In the next section, we describe a method to determine the local anisotropy from low field SHMR data.

5 Extraction of local anisotropy

The longitudinal SHMR takes the form [21]

$$\rho_{\text{long}} = \rho_0 + \Delta\rho_{\text{SHMR}}(1 - m_y^2), \tag{E5}$$

where m_y is the y component of the magnetic moment unit vector $\mathbf{m} = \mathbf{M}/|\mathbf{M}|$ (geometry shown in inset of Fig. S7 (a)). When $|\mathbf{H}|$ is much larger than the anisotropy, $\mathbf{m}$ always aligns with $\mathbf{H}$ and thus SMHR follows a $\cos^2\beta$ dependence. However, $\mathbf{m}$ and $\mathbf{H}$ no longer align for low values of $|\mathbf{H}|$, resulting in a deviation from $\cos^2\beta$. This can be seen in Fig. S7 (a) where we measure SHMR with $\mu_0 H = 50$ mT. To analyze this deviation, the equilibrium angle of $\mathbf{m}$ must be determined by minimizing the free energy [23]

$$F = -\mu_0 \mathbf{H}\cdot\mathbf{M} + \frac{1}{2}\mu_0 M_s \left[M_{\text{eff}} \cos^2\beta_M - \frac{1}{8} H_{\text{cub}}(3 + \cos 4\phi)\sin^4\beta_M \right], \tag{E6}$$

where β_M is the angle $\mathbf{M}$ makes with z, ϕ is the in-plane angle $\mathbf{H}$ makes with the [100] axis, and H_{cub} is the in-plane cubic magnetocrystalline anisotropy. For SHMR measurements we have $\phi = 0$. Let β be the angle $\mathbf{H}$ makes with z. Setting $\partial F/\partial\beta_M = 0$ yields

$$r_1 \sin(\beta_M - \beta) - \frac{1}{2}\sin(2\beta_M) - 2r_2 \sin^3\beta_M \cos\beta_M = 0, \tag{E7}$$

where $r_1 = |\mathbf{H}|/M_{\text{eff}}$ and $r_2 = H_{\text{cub}}/M_{\text{eff}}$. We numerically solve equation (E7) for β_M, and use $m_y = \sin\beta_M$ in equation (E5) to fit the SHMR data. From this fit we can extract r_1 and r_2, from which we obtain M_{eff} (from r_1) and subsequently H_{cub} (from r_2). This fit is shown as the red line in Fig. S7 (a), giving $\mu_0 M_{\text{eff}} = -28.7 \pm 0.5$ mT and $\mu_0 H_{\text{cub}} = -0.19 \pm 0.04$ mT. The value of $|\mu_0 M_{\text{eff}}|$ is in excellent agreement with the peak half-width of the red trace in Fig. S6 (b).

Joule heating has been speculated as an alternative mechanism for changing the anisotropy [24]. To investigate this, we performed low field ($\mu_0 H = 50$ mT) SHMR measurements for various measuring currents. The extracted M_{eff} and H_{cub} values are shown in Fig. S7 (b) and (c). No appreciable dependence on measuring current is observed, suggesting that Joule heating does not play a major role in changing the anisotropy in our system.

6 X-ray absorption spectroscopy (XAS) and X-ray magnetic circular dichroism (XMCD)

Fig. S8 (a) and (b) show the XAS and XMCD spectra of a typical LAFO film at the Fe L_3 and L_2 edges. The XMCD spectra is defined as the difference between the XA spectra with the magnetization anti-parallel and parallel to the circular polarization vector of the X-ray beam. In the XMCD spectra, the negative peak at $E \approx 710$ eV correspond to Fe^{3+} with octahedral coordination ($Fe^{3+}_{O_h}$), whereas the positive peak at $E \approx 709$ eV correspond to Fe^{3+} with tetrahedral coordination ($Fe^{3+}_{T_d}$). The signs of these two peaks indicate that the moments from $Fe^{3+}_{T_d}$ and $Fe^{3+}_{O_h}$ align anti-parallel and parallel with the field respectively, which is consistent with the antiferromagnetic superexchange among $Fe^{3+}_{T_d}$ and $Fe^{3+}_{O_h}$ in LFO [25]. The red trace in Fig. S8 (b) is

a fit using a linear combination of two reference spectra shown in Fig. S8 (c). The experimental data is well accounted for with only the presence of $Fe^{3+}_{T_d}$ and $Fe^{3+}_{O_h}$ ions, without inclusion of cation disorder such as the presence of Fe^{2+}. This indicates that the primary magnetic ion in LAFO is Fe^{3+} and cation disorder is minimal.

7 Spin orbit torque switching of thicker films

In addition to the SOT switching data on the 4 nm LAFO film shown in the manuscript, we also performed SOT switching on a thicker 15 nm LAFO film as shown in Fig. S9. These thicker films have a much larger anisotropy and therefore a larger current is required for switching. The critical switching current J_c is about 21×10^6 A/cm^2 and 17.7×10^6 A/cm^2 for in-plane fields of 10 mT and 50 mT respectively. These values are similar to those reported in YIG systems [24, 26, 27]. However, LAFO provides an advantage in that its anisotropy can be lowered for thinner samples, both of which reduce J_c, allowing for record low critical switching currents at low thicknesses.

References

[1] Emori, S. *et al.* Ultralow damping in nanometer-thick epitaxial spinel ferrite thin films. *Nano Lett.* **18**, 4273–4278 (2018).

[2] Budhani, R. C. *et al.* Pseudomorphic spinel ferrite films with perpendicular anisotropy and low damping. *Appl. Phys. Lett.* **113**, 082404 (2018).

[3] Zheng, X. Y., Riddiford, L. J., Wisser, J. J., Emori, S. & Suzuki, Y. Ultra-low magnetic damping in epitaxial $Li_{0.5}Fe_{2.5}O_4$ thin films. *Appl. Phys. Lett.* **117**, 092407 (2020).

[4] Dionne, G. F. Magnetic-anisotropy and magnetostriction constants of substituted lithium ferrites at 300 K. *J. Appl. Phys.* **40**, 4486–4490 (1969).

[5] Mazen, S. A. & Elmosalami, T. A. Structural and elastic properties of Li-Ni ferrite. *ISRN Condens. Matter Phys.* **2011**, 820726 (2012).

[6] Cullity, B. D. & Graham, C. D. *Magnetostriction and the Effects of Stress*, chap. 8, 241–273 (John Wiley & Sons, Ltd, 2008).

[7] Wisser, J. J. *et al.* Ultrathin interfacial layer with suppressed room temperature magnetization in magnesium aluminum ferrite thin films. *Appl. Phys. Lett.* **115**, 132404 (2019).

[8] Mitra, A. *et al.* Interfacial origin of the magnetisation suppression of thin film yttrium iron garnet. *Sci. Rep.* **7**, 11774 (2017).

[9] Zakeri, K. *et al.* Spin dynamics in ferromagnets: Gilbert damping and two-magnon scattering. *Phys. Rev. B* **76**, 104416 (2007).

[10] Farle, M. Ferromagnetic resonance of ultrathin metallic layers. *Rep. Prog. Phys.* **61**, 755–826 (1998).

[11] Chappert, C., Dang, K. L., Beauvillain, P., Hurdequint, H. & Renard, D. Ferromagnetic resonance studies of very thin cobalt films on a gold substrate. *Phys. Rev. B* **34**, 3192–3197 (1986).

[12] Cui, Y. *et al.* Interfacial perpendicular magnetic anisotropy and damping parameter in ultra thin co2feal films. *Appl. Phys. Lett.* **102**, 162403 (2013).

[13] Johnson, M. T., Bloemen, P. J. H., den Broeder, F. J. A. & de Vries, J. J. Magnetic anisotropy in metallic multilayers. *Reports on Progress in Physics* **59**, 1409–1458 (1996).

[14] Wen, Z., Sukegawa, H., Mitani, S. & Inomata, K. Perpendicular magnetization of Co_2FeAl full-heusler alloy films induced by MgO interface. *Appl. Phys. Lett.* **98**, 242507 (2011).

[15] Gowtham, P. G., Stiehl, G. M., Ralph, D. C. & Buhrman, R. A. Thickness-dependent magnetoelasticity and its effects on perpendicular magnetic anisotropy in Ta/CoFeB/MgO thin films. *Phys. Rev. B* **93**, 024404 (2016).

[16] Jiang, S. *et al.* Ferromagnetic resonance linewidth and two-magnon scattering in $Fe_{1-x}Gd_x$ thin films. *AIP Adv.* **7**, 056029 (2017).

[17] Luo, C. *et al.* Enhancement of magnetization damping coefficient of permalloy thin films with dilute Nd dopants. *Phys. Rev. B* **89**, 184412 (2014).

[18] Riddiford, L. J. *et al.* Efficient spin current generation in low-damping Mg(Al, $Fe)_2O_4$ thin films. *Appl. Phys. Lett.* **115**, 122401 (2019).

[19] Nakayama, H. *et al.* Spin Hall magnetoresistance induced by a nonequilibrium proximity effect. *Phys. Rev. Lett.* **110**, 206601 (2013).

[20] Althammer, M. *et al.* Quantitative study of the spin Hall magnetoresistance in ferromagnetic insulator/normal metal hybrids. *Phys. Rev. B* **87**, 224401 (2013).

[21] Chen, Y.-T. *et al.* Theory of spin Hall magnetoresistance. *Phys. Rev. B* **87**, 144411 (2013).

[22] Amamou, W. *et al.* Magnetic proximity effect in Pt/$cofe_2o_4$ bilayers. *Phys. Rev. Mater.* **2**, 011401 (2018).

[23] Gray, M. T. *et al.* Spin-current generation in low-damping $ni_{0.65}zn_{0.35}al_{0.8}fe_{1.2}o_4$ spinel ferrite. *Phys. Rev. Appl* **9**, 064039 (2018).

[24] Avci, C. O. *et al.* Current-induced switching in a magnetic insulator. *Nat. Mater.* **16**, 309–314 (2017).

[25] White, G. & Patton, C. Magnetic properties of lithium ferrite microwave materials. *J. Magn. Magn. Mater.* **9**, 299–317 (1978).

[26] Guo, C. Y. *et al.* Spin-orbit torque switching in perpendicular $Y_3Fe_5O_{12}$/Pt bilayer. *Appl. Phys. Lett.* **114**, 192409 (2019).

[27] Ding, J. *et al.* Nanometer-thick yttrium iron garnet films with perpendicular anisotropy and low damping. *Phys. Rev. Appl.* **14**, 014017 (2020).

[28] Brice-Profeta, S. *et al.* Magnetic order in γ-Fe_2O_3 nanoparticles: A xmcd study. *J. Magn. Magn. Mater.* **288**, 354–365 (2005).

[29] Kim, J.-Y., Koo, T. Y. & Park, J.-H. Orbital and bonding anisotropy in a half-filled $gafeo_3$ magnetoelectric ferrimagnet. *Phys. Rev. Lett.* **96**, 047205 (2006).

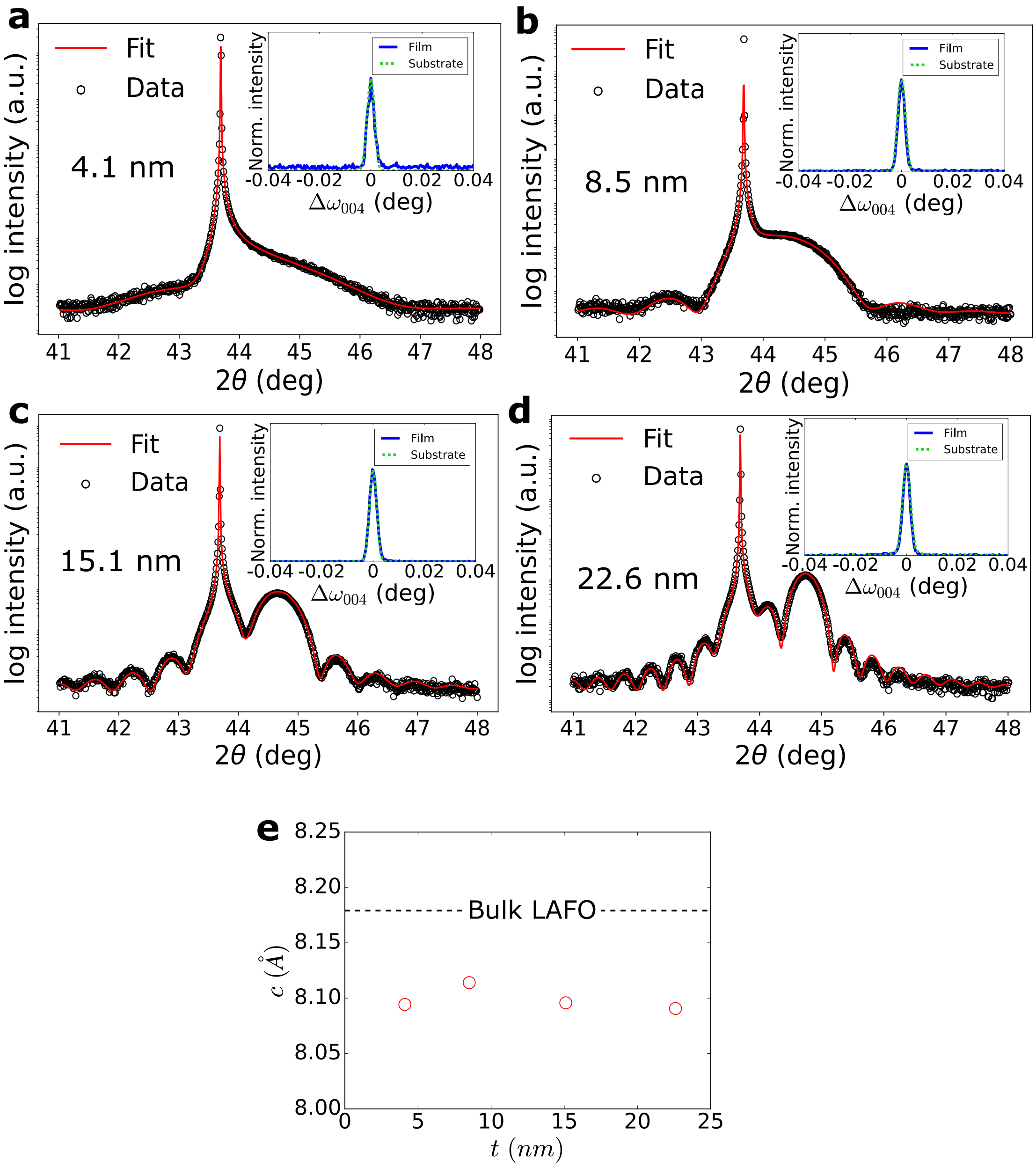

Figure S 1: X-ray diffraction structural characterization of LAFO films. (a)-(d) X-ray diffraction symmetric scans of the (004) peak for four different LAFO film thicknesses with the solid red lines as a fit to the data (details described in the text). The insets show the (004) rocking curves of the film and substrate. (e) Out-of-plane lattice constant c of the LAFO films across thicknesses extracted from the fits to the XRD data. The error bars are smaller than the symbol size.

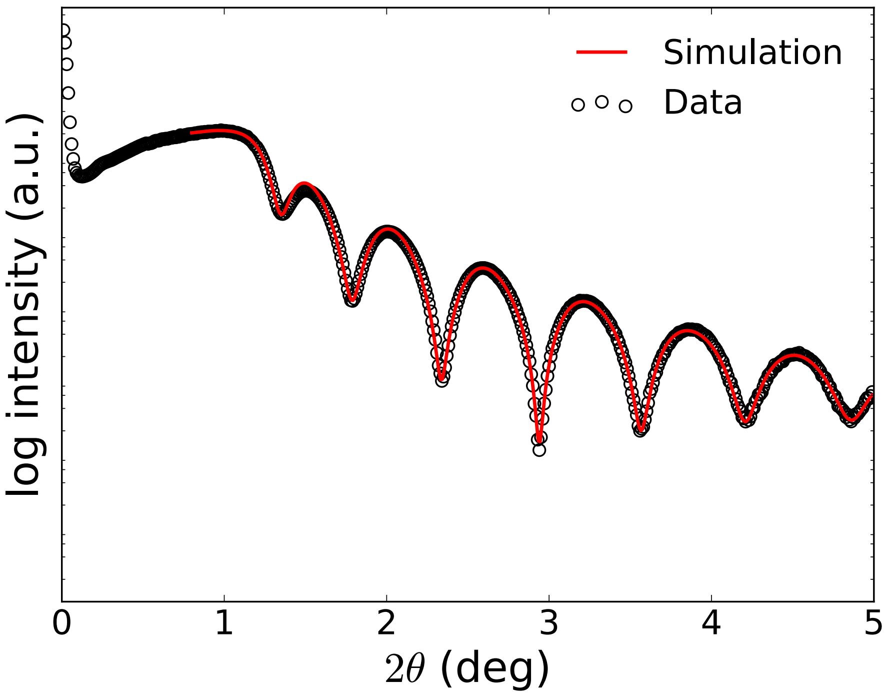

Figure S 2: X-ray reflectivity of a Pt(15 nm)/LAFO(15 nm)/MGO sample, showing pronounced Kiessig fringes.

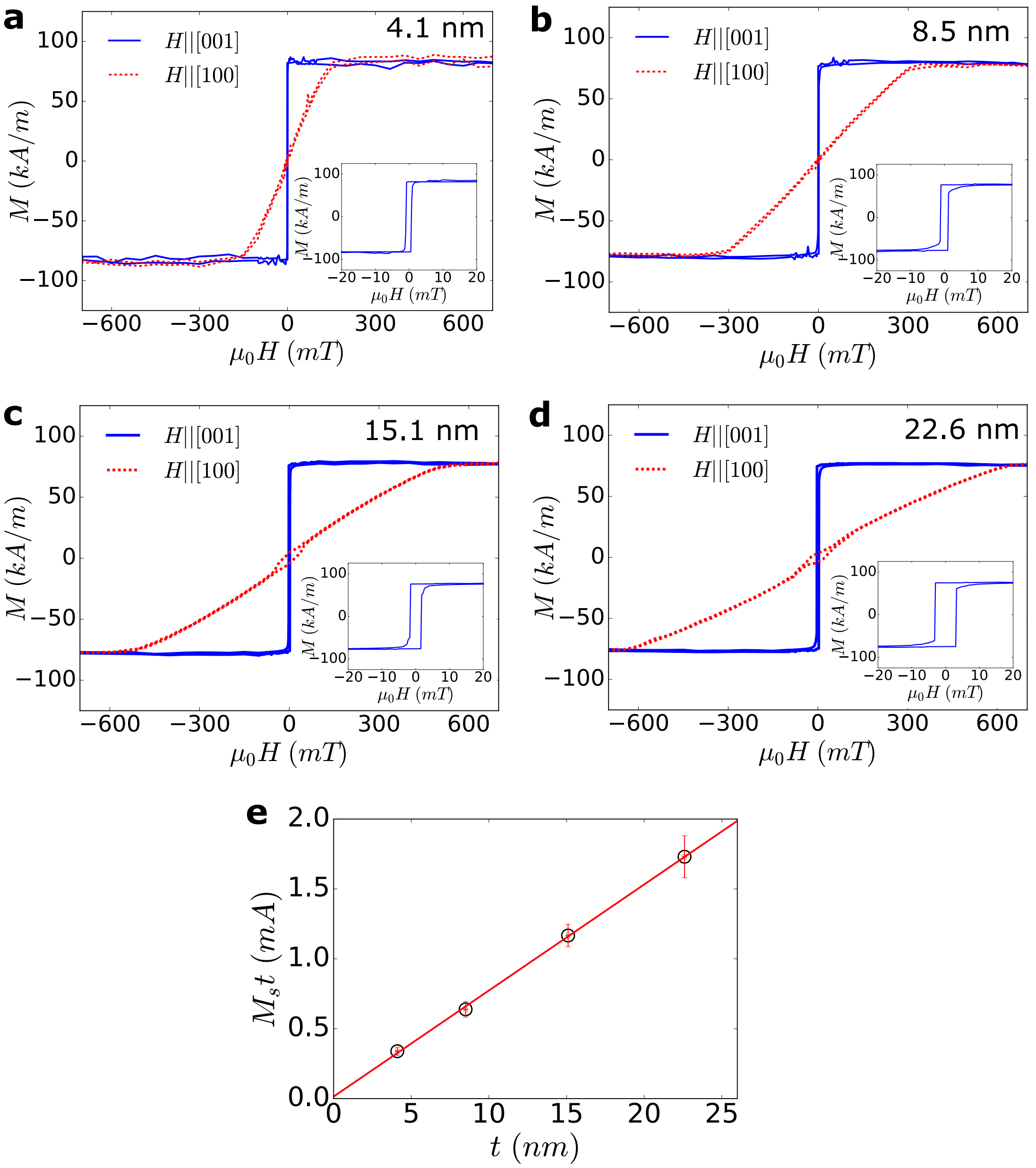

Figure S 3: SQUID magnetometry of LAFO films at room temperature. (a)-(d) Magnetization as a function of applied field measured using SQUID magnetometry for the four LAFO film thicknesses. All four thicknesses exhibit PMA, albeit with decreasing strength for thinner films. The insets show a detailed view of the [001] trace near the origin. (e) Net saturation moment per unit area as a function of thickness t. The red line is a linear fit yielding a y intercept of 0.01 ± 0.02 mA. The error bars on the data points were estimated based on the uncertainty in the saturation moment, t, and the film area.

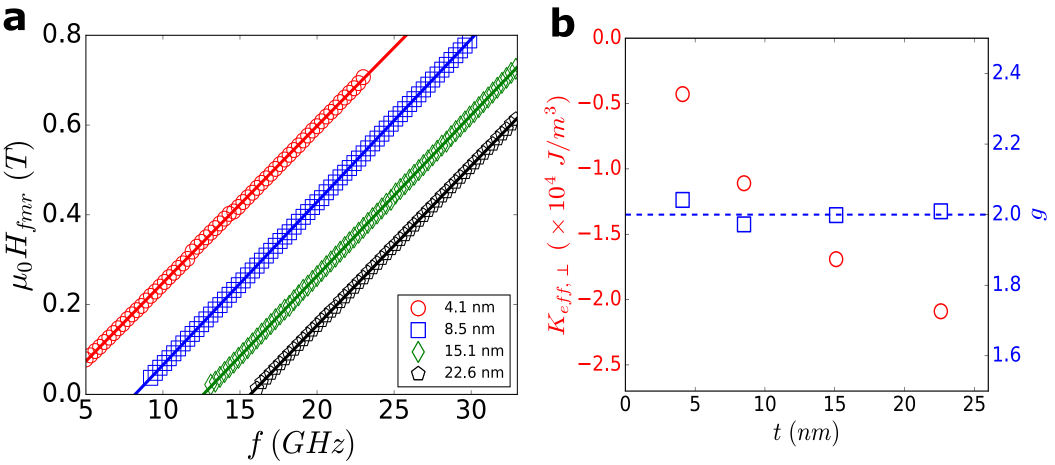

Figure S 4: Anisotropy and g factor characterization of LAFO films. (a) FMR resonance field H_{fmr} as a function of frequency f for the four LAFO film thicknesses. The solid lines are linear fits to equation (2) in the main text, allowing the extraction of M_{eff} and g. (b) Effective out-of-plane uniaxial anisotropy energy density $K_{\text{eff},\perp} = \mu_0 M_{\text{eff}} M_s/2$ and g across thicknesses. The horizontal blue dotted line is at $g = 2.0$. The error bars are smaller than the symbol size.

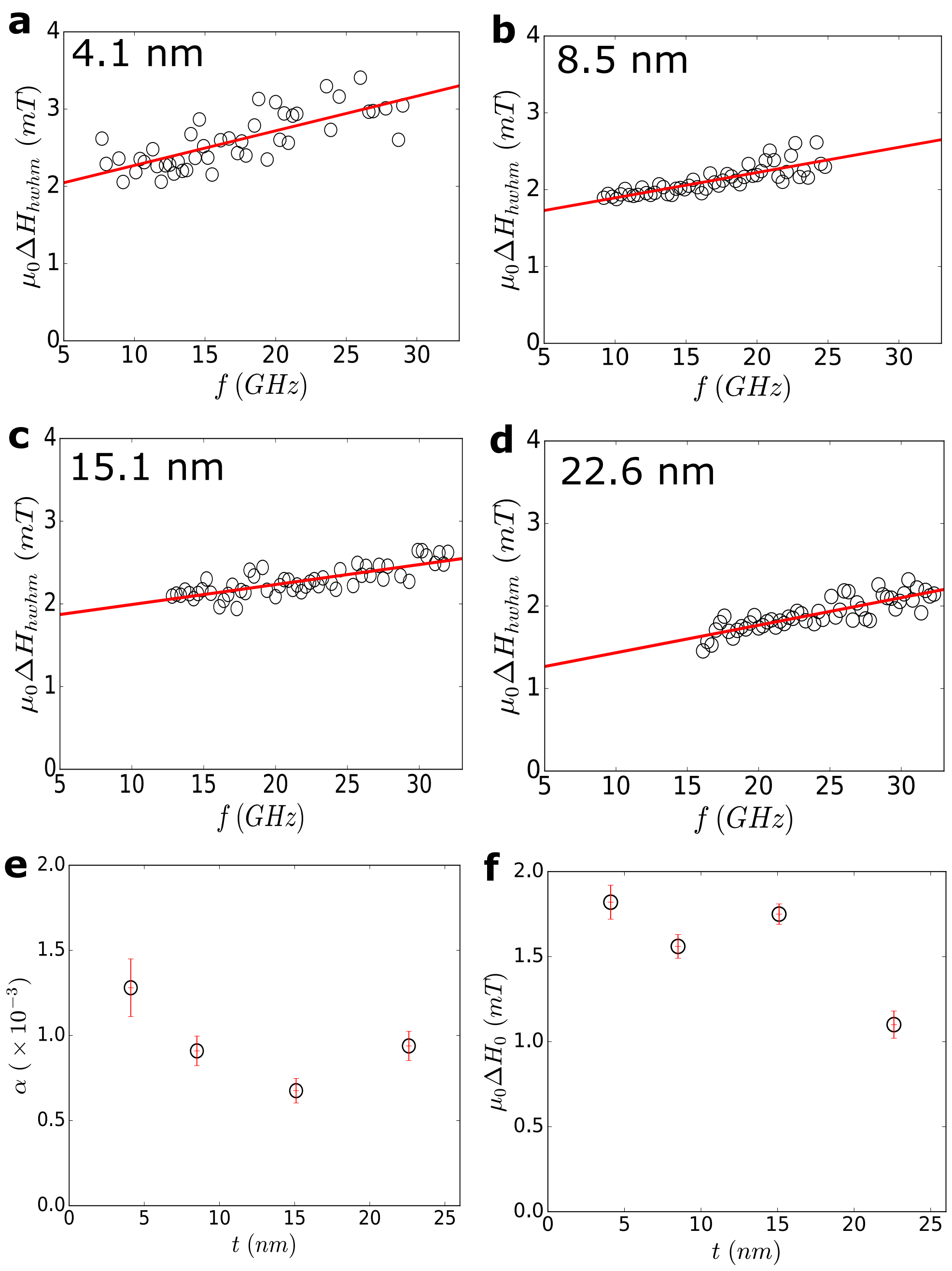

Figure S 5: Damping characterization of LAFO films (a)- (d) FMR linewidth $\mu_0 \Delta H_{\mathrm{hwhm}}$ as a function of frequency f for the four LAFO film thicknesses. The red lines are linear fits to equation (1) in the main text. (e) Extracted Gilbert damping α across thicknesses. The error bars represent one sigma deviation from the slope of the linear fit. (f) The y intercept of the linear fits from (a) - (d), representing the inhomogenous broadening ΔH_0. The error bars represent one sigma deviation from the intercept of the linear fit.

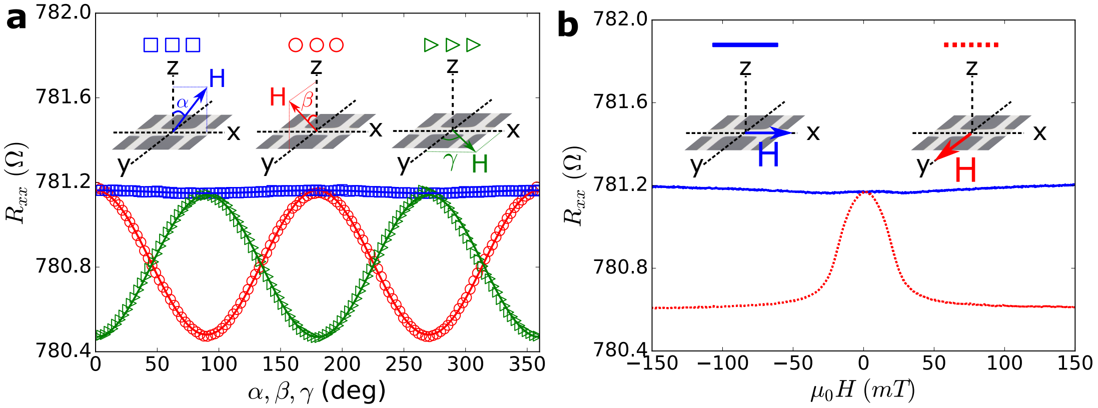

Figure S 6: Angular dependent magnetoresistance on the patterned Pt(2 nm)/LAFO(4.1 nm)/MGO. (a) AMR (blue squares), SHMR (red circles), and in-plane MR (green triangles) with $\mu_0 H = 1$ T. The inset shows the field orientations for each measurement. The open circles are data, and the solid lines are fits to a cosine squared lineshape. (b) Longitudinal magnetoresistance with H parallel (blue solid line) and perpendicular (red dashed line) to the current in-plane.

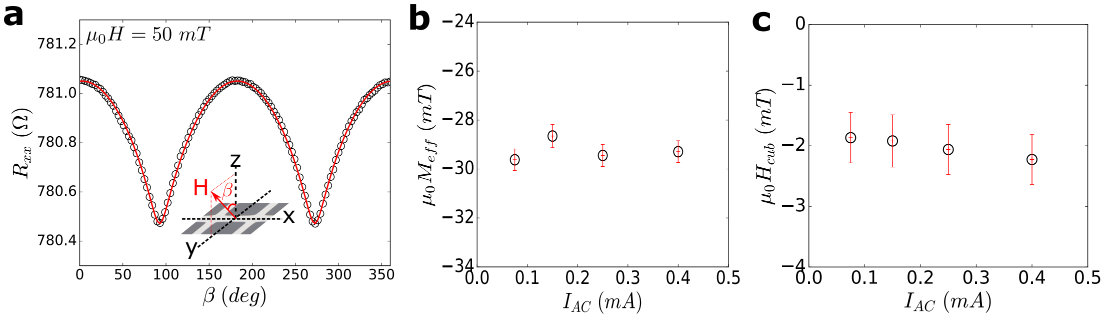

Figure S 7: Low field SHMR measurements on the patterned Pt(2 nm)/LAFO(4.1 nm)/MGO. (a) SHMR measurement at $\mu_0 H = 50$ mT and a measuring current of 0.15 mA, showing deviation from the usual cosine squared behavior. The red line is a fit as described in the text. (b)(c) Extracted M_{eff} and H_{cub} from the SHMR fit at 50 mT for various longitudinal currents.

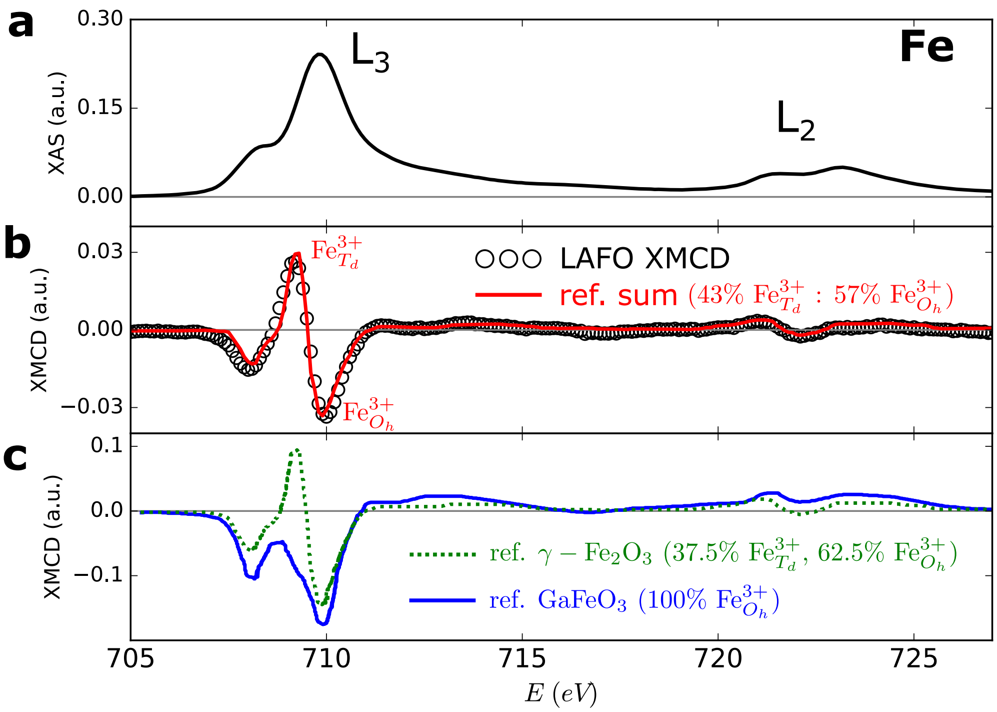

Figure S 8: XAS and XMCD measurements of a typical LAFO film. (a) XAS on the Fe L_3 and L_2 edges of LAFO. (b) XMCD of LAFO along with a fit to a linear combination of two reference traces shown in (c). (c) XMCD reference traces of $\gamma - Fe_2O_3$ [1, 28] and $GaFeO_3$ [1, 29], both of which contain only Fe^{3+} ions.

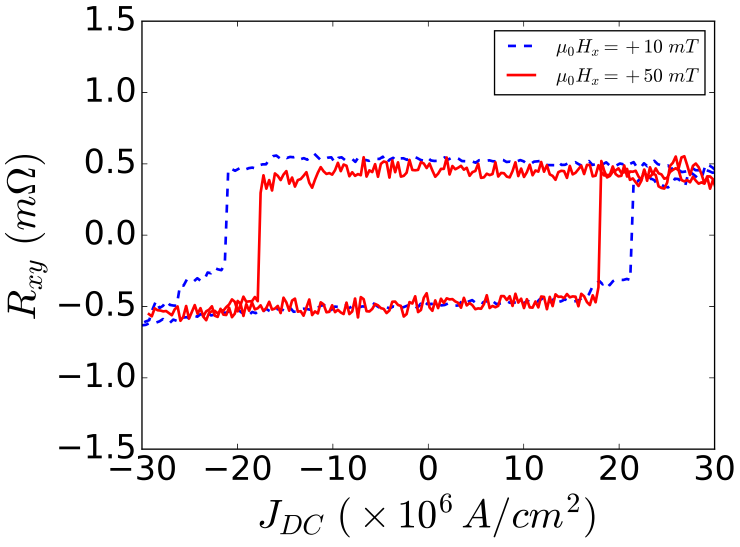

Figure S 9: Spin orbit torque switching for a Pt(5 nm)/LAFO(15 nm)/MGO sample for in-plane fields of 10 mT and 50 mT.
